# Exclusion of married adolescents in a study of gestational diabetes mellitus: a case study

**DOI:** 10.1186/s12978-017-0423-1

**Published:** 2017-12-14

**Authors:** Mala Ramanathan, K. Sakeena

**Affiliations:** 1 AMCHSS, Sree Chitra Tirunal Institute for Medical Sciences and Technology, Trivandrum, India; 2 Kerala Health Services, Kerala, India

**Keywords:** Gestational diabetes mellitus, Adolescent women, Unfair exclusion, Confidentiality, Mature minors

## Abstract

A study on gestational diabetes mellitus (GDM) among 200 married women in Malappruam, Kerala, India, chose to exclude married women below the age of 18 from participation. Marriages before age 18 are not considered legally valid and persons below age 18 do not have the status of an adult. Parents are considered the legal guardians of married women under age 18, but because marriages are patrilocal, obtaining consent from parents would have time costs. Further, obtaining parental consent may also be considered disrespectful of the in-laws. The inclusion of married adolescents in this study was considered difficult for these reasons. This exclusion can also result in wrongly estimating the levels of GDM among all women at risk. We argue that such exclusion is also unethical; it unfair to exclude women who stand to benefit from participation by enabling them to identify the enhanced life time risk for diabetes mellitus and monitor their future health status better. Recognizing married adolescents as emancipated minors would enable their participating without violating confidentiality regarding their GDM status to parents and in-laws.

## Case background

According to the District Level Household Surveys 4, which provide reproductive and child health-related data up to the district level in India, 24.7% of adult women aged ≥ 18 in Kerala reported blood sugar levels > 140 mg/dl and 13.5% reported blood sugar levels > 160 mg/dl [1]. For the district of Malappuram (within the state of Kerala), 14% of the women aged ≥ 18 reported blood sugar levels > 140 mg/dl and 7.1% reported levels > 160 mg/dl [2]. The burden of diabetes mellitus among adult women motivated a public health study on post-partum diabetes screening and follow-up for women with gestational diabetes mellitus (GDM) in the district of Malappuram (K Sakeena: Patterns of and factors associated with postpartum diabetes screening in women diagnosed with gestational diabetes mellitus in Malappuram district, unpublished).

The primary objectives of this study were to examine the patterns of and factors associated with post-partum diabetes among women who had GDM during their most recent pregnancy and to document post-partum morbidity among these women. A secondary objective was to understand health providers’ perspectives on appropriate follow-up care for patients who had experienced GDM.

The study design used a mixed methods approach that included (i) a cross-sectional survey with a structured interview schedule for the GDM patients and (ii) in-depth interviews (using an interview guide) for health providers. The sample size for the study was 200 married women diagnosed with GDM during their most recent pregnancy in selected hospitals. The women included in the study were to have delivered three to 6 months before the date on which the survey was administered to ensure that all participants had a minimum of 9 weeks of post-partum experience to include in the morbidity study. Adolescent married women were excluded from participation due to researcher concerns that parental consent would be difficult to obtain and that obtaining parental consent would be considered disrespectful of the in-laws.

## Ethical discussion

### Unfair exclusion criteria

The study on GDM in Malappuram district chose to exclude married women below age 18 from participation. This pragmatic approach to the research unfairly excluded a group of women and their children who are particularly vulnerable to getting diabetes mellitus. These young women and their children would benefit from screening and advice on lifestyle modification to manage or to prevent diabetes mellitus. The original study was designed to include 200 married women of all ages diagnosed with GDM during their most recent pregnancy in selected hospitals. The inclusive age range was important as GDM can affect all women who get pregnant and, in India, pregnancy generally tends to follow marriage. Other studies have shown that the risk of GDM follows the risk of type 2 diabetes mellitus in most populations and is associated with higher maternal age. Such risk among Asians was higher in the United States and Europe [3]. As the risk of pregnancy among women in younger ages is lower in North America and Europe, it is possible that reported prevalence of GDM could also be affected by the smaller share of pregnant women in younger ages. Even so, two out of every 100 teenaged pregnant women did develop GDM in the United States, and this risk is highest among Asian/Pacific Islander adolescent women when compared to non-Hispanic whites [4]. Therefore, including women of all ages within the reproductive span is extremely important as a matter of fairness.

Despite this strong rationale, the study team faced significant challenges with the recruitment of married women under the age of 18 because such marriages are not legally recognized. In India, the *Prohibition of Child Marriage Act* stipulates that the legal age for marriage is 18 for females and 21 for males [5]. In the state of Kerala—a state known for high levels of literacy in general (and female literacy in particular), better access to healthcare, and relatively higher ages at marriage—the prevalence of child marriage involving females under the age of 18 was 2.8% in 2012–13 [1]. However, in the Malappuram district–the most densely populated of the 14 districts of Kerala and the district with the highest population growth rates–the prevalence of child marriage involving adolescent girls under the age of 18 was 26.3% during this same time period and the percentage of all births in Malappuram to women between the ages of 15–19 years in 2012–13 was 6.2% [2].

### Consent for emancipated minors

Typically, participation in research requires informed consent from adults (and emancipated minors) and assent from minors. In India, persons below the age 18 are not considered legal adults and the concept of emancipated minor is not legally recognized [6]. For this reason, research involving married adolescent girls is difficult with regard to consent.

Current practice in India is that ethics committees require the assent of married adolescent girls and the consent of their legal guardians [7]. Legal guardians of females below age 18 are nominally their parents. The problem with obtaining consent from the parents is that after the marriage, most girls move to their affinal homes and live with their husbands and in-laws. Neither the husband (assuming he is above the age of 18) nor the in-laws are recognized as legal guardians.

These practical difficulties involved in obtaining consent from the legal guardians (the parents) lead to the exclusion of married women below age 18 in the study. Seeking consent from the parents was a legal option, but might have been seen as disrespectful of the marriage (even though the law does not recognize the marriage of girls below age 18). Moreover, attempting to obtain such consent was not without a time cost. Consent from parents or in-laws would also violate confidentiality requirements for the young women as the purpose of the study would have to be mentioned to those giving consent.

## Conclusions

Studies on the reproductive health of adolescents require that confidentiality be respected, even if consent for participation is obtained from parents or legal guardians [8]. Ethics committees in India have allowed for a young adolescent to identify an adult living in her household whom she identifies as having her welfare at heart to provide consent on her behalf (Vivek RV: Healthcare seeking behaviour among female adolescents for reproductive morbidities in the urban slums of Chandigarh, unpublished). Making such an allowance for studies among married adolescents, particularly when they will directly benefit from such participation—as in the case of GDM where knowledge of the condition would enable the women to better participate in follow-up care—is one possible solution to this unfair exclusion. Such a policy has implications for studies elsewhere in India where the proportion of women married before age 18 is likely to be even higher than that found in Malappuram. In such circumstances, exclusions due to the difficulties in overcoming cultural barriers would affect the outcome measure and also exclude far more women than would happen in situations where marriages before age 18 are fewer in number. However, this solution would not respect confidentiality requirements.

Alternatively, if there is recognition of a married adolescent as a mature minor, this form of unfair exclusion can be avoided. In low- and middle-income countries, where married adolescents and/or pregnant adolescents are most likely to be found and benefit from engagement in research, lack of such recognition is problematic and often unfair. Recognition of a married adolescent as a mature minor would prevent this form of unfair exclusion and also serve to address issues relating to maintaining confidentiality regarding the young woman’s GDM status.

## Abbreviations

GDM: Gestational diabetes mellitus
